# Urinary incontinence among women in sub-Saharan Africa – an overview

**DOI:** 10.3389/fruro.2023.1289421

**Published:** 2023-11-08

**Authors:** Chidimma A. Omeke, Charles E. Azuka

**Affiliations:** ^1^ Department of Obstetrics & Gynaecology, Enugu State University of Science and Technology, Enugu, Nigeria; ^2^ Selazona Consulting LLC, Liberty Township, OH, United States

**Keywords:** urinary incontinence, sub-Saharan Africa, obstetric fistula, pelvic floor, non-fistulous incontinence, Colpo suspension, urge incontinence, diapers

## Abstract

Urinary Incontinence, the uncontrolled or involuntary loss of urine, is a prevalent condition among women that is frequently underdiagnosed and underreported, particularly in sub-Saharan Africa (SSA). The social stigma attached to urinary incontinence contributes to low presentation rates for care, and the help-seeking behavior of SSA women with urinary incontinence in this region. Thus, the occurrence of urinary incontinence imposes a considerable burden on women in SSA, leading to physical, social, and psychological complications. A multitude of challenges, such as limited availability of urogynaecological facilities, corruption, etc, have collectively contributed to the scarcity of information on how to manage urinary incontinence in this region. The commonplace management of urinary incontinence is the use of adult diapers, mostly among older women in this region. While it is essential to carry out more research to comprehend the impact of urinary incontinence on women in Sub-Saharan Africa and to promote awareness of medical and surgical interventions; It is recommended that the governing bodies of these regions enhance their endeavors to provide essential facilities and training a skilled workforce to manage this condition at a subsidized cost.

## Introduction

Urinary Incontinence (uncontrolled or involuntary loss of urine) is a condition that is relatively common among women, yet it is often underdiagnosed and underreported, particularly in sub-Saharan Africa (SSA). A review article including 30 different studies on prevalence and risk factors of pelvic organ prolapse and incontinence in developing countries revealed a mean prevalence of urinary incontinence of 28.7%, with a wide range of 5.2 – 70.8% (1).

Urinary incontinence (UI) can be classified into two types: non-fistulous and fistulous urinary incontinence. The incidence of non-fistulous incontinence, which includes stress incontinence, urge incontinence, and mixed urinary incontinence, is frequently associated with various risk factors, including high parity, vaginal and assisted delivery, number of abortions, constipation, obesity, menopause, recurrent urinary tract infection, hysterectomy, foetal weight, aging, and comorbid conditions such as diabetes and chronic pulmonary disease. Vesicovaginal fistula can cause incontinence, which is often associated with obstetric causes, particularly obstructed labour (2). Though not life-threatening, urinary incontinence can negatively impact the psychological, physical, and social well-being of the affected individual. The aim of this paper is to give a general overview of urinary incontinence in Africa with its associated management challenges, while identifying knowledge gaps that require further research.

## Occurrence and impact

The occurrence of urinary incontinence imposes a considerable burden on women in SSA, leading to physical, social, and psychological complications. A cross-sectional study consisting of quantitative and qualitative components was conducted in seven administrative regions of rural Ethiopia. Fifty-two women with obstetric fistula were interviewed on the subjects of their socioeconomic status and history of the development of obstetric fistula. According to the study, obstetric fistula ha a significant impact on the mental health and family life of affected women, as evidenced by the fact that 92.3% of respondents reported experiencing varying degrees of depression, while 53.8% had suicidal ideation (3). In addition, the report showed that 69.2% of women had been divorced, and 19.2% were forbidden from having meals with their family members (3). Non-fistulous urinary incontinence can lead to limitations in physical activities, such as housework, social activities, and sexual intercourse. It may also lead to feelings of isolation, shame, embarrassment, frustration, depression, annoyance, and uncleanliness (3).

The management of urinary incontinence can be more costly than expected, considering the expenses involved in obtaining routine care items such as medications, surgical interventions, behavioral therapy, devices, bladder pads and diapers and diagnostic care such as laboratory tests. Studies conducted in developed nations such as the United States have revealed that urinary incontinence is linked to a significant economic burden from both a societal and patient perspective (4). One such study established that the condition is highly costly, comparable to other chronic diseases that affect women (5). Another study showed that women who experience severe urinary incontinence incur an annual expenditure of $900 for incontinence routine care, and this condition causes a significant decrease in health-related quality of life (6).

In the SSA region, a multitude of challenges, such as limited availability of urogynecological facilities, high incidence of infectious diseases, decreased life expectancy, poverty, corruption, inadequate distribution of health resources, human rights violations, and insufficient empowerment of women regarding their reproductive health, have collectively contributed to the scarcity of information on how to manage urinary incontinence in this region.

The social stigma attached to urinary incontinence contributes to low presentation rates for care, particularly in SSA. A study conducted in 2010 investigated the incidence and associated triggering factors of urinary incontinence among 5,000 black women in SSA. The findings showed that stress incontinence was the most prevalent, followed by urge incontinence, with a prevalence rate of 2.8% (7). The results of another study, which examined the help-seeking behavior of 139 women with urinary incontinence, showed that a mere 18 of them (12.9%) sought help. Some respondents had no reason for not seeking hospital care. The most cited reason for not seeking hospital care among respondents was the belief that their condition was not life-threatening (comprising 51.2% of the responses). Additionally, 18.2% of respondents were doubtful about the availability of treatment for their condition. Other reasons were lack of funds, shyness, and fear of complications (8).

The help-seeking behavior of SSA women with urinary incontinence may be further influenced by other factors, such as lack of awareness, shortage of specialists, absence of health insurance resulting in high out-of-pocket expenses for medical care, sociocultural restrictions, insufficient access to quality healthcare, and the prevalence of traditional medical practices. The integration of traditional medicine in the SSA region may be credited to the persuasive endorsements of non-orthodox practitioners who advocate for complementary alternative medicine as a cure for all illnesses, and prompt patients to experiment with it. In Nigeria, for example, before introducing orthodox medicine, herbal medicines were the centerpiece of treatment for most ailments, and traditional herbalists cultivated and dispensed preparations (9). The poor economic state and the rising costs of orthodox medicines have resulted in a surge in using perceived cheaper herbal medications to treat incontinence. The administration of herbal therapies is frequently aligned with the cultural beliefs of the individuals. An Ethiopian community-based study showed that 46.1% of the women believed that evil spirits, curses, or sins caused the development of obstetric fistula. Some other communities saw vesicovaginal fistula as a consequence of immoral sexual behavior or witchcraft^3.^

## Current treatment options

The treatment of non-fistulous UI can be surgical or non-surgical, depending on the type of incontinence, clinical manifestations, the patient’s inclination for treatment, the readiness to undergo invasive procedures, and the treatment’s expenses.

Most women often experience improvement with the non-surgical approach, which is usually the first line of management and is less invasive, with cure rates of 10% - 15%. This includes behavioral changes, lifestyle modification, pelvic floor retraining, and use of mechanical devices. The response depends on the patient’s compliance. This treatment is commonly suggested for patients who have a desire for future pregnancy, cannot undergo surgical procedures because of medical reasons, or opt out of surgery (10).

While the surgical approach presents greater invasiveness and the potential for complications like urge incontinence and dysfunctional voiding, it boasts a higher cure rate and is less reliant on the patient’s compliance (11).

Pelvic floor exercises and subsequent monitoring are frequently utilized for women who seek medical attention for mild stress incontinence. In contrast, individuals who display symptoms of overactive bladder, overflow incontinence, and other UI causes are given medication and, if deemed necessary, catheterization. Counselling plays a pivotal role in assisting these women to overcome the stigma of incontinence and promoting future adherence to treatments and health-seeking behavior (1).

Further management of urinary incontinence can be achieved through the use of intravaginal devices, which include short and long tampons, pessaries, continence rings, and tampon-like devices, as well as urethral devices such as intraurethral plugs and urethral patches. However, these devices are barely accessible, particularly in hospitals owned by the government, which is the most prevalent source of healthcare in most of the SSA region (12).

The management of urinary incontinence through surgical means involves various options such as anterior colporrhaphy with or without Kelly/Pacey suture, urethrocliesis, usage of urethral bulking agents, retropubic mid-urethral tape procedures, trans-obturator mid-urethral tape procedures, Marshall–Marchetti–Krantz procedure and Burch Colpo suspension (11). The surgeries are highly specialized and are performed only by urogynaecologist, who unfortunately are not evenly distributed and are in short supply in SSA.

Hence within the context of the insufficient healthcare services offered in low- and middle-income countries in SSA, the government’s prioritization of urinary incontinence treatments is low (10). Consequently, it has become commonplace for adult diapers to be utilized to manage urinary incontinence, particularly among older women in SSA. The utilization of diapers is a more viable option for women who encounter urinary incontinence to manage their condition. These diapers are easily obtainable in the market and do not require a distinctive prescription beforehand. However, these diapers are also expensive, further adding to the financial burden of managing urinary incontinence.

## Management of urinary incontinence: the role of the government

The government remains a major provider of health care in most of the SSA region, and thus her role in the management of urinary incontinence cannot be overemphasized. Despite the fact that the complex interactions between a country’s medical, social, economic, and environmental factors could affect the management of UI, there are practical steps that the government health institutions could take to address the problems of UI, including:

### Integrating adult incontinence management into existing healthcare programs

Present in many countries are programs that address maternal and child health and women-focused programs. Such programs can train healthcare workers, including nurses and community health workers, to provide proper guidance and support to individuals with incontinence. The government at the institutional level should promote continuing education for healthcare professionals on the latest advancements in incontinence management.

In addition, to basic routine medical supplies, it is imperative that government institutions ensure a continuous and affordable supply of incontinence products, including diapers and pads, particularly for low-income individuals and communities. Government can explore partnerships with manufacturers of incontinence products to lower costs and improve distribution and, where possible, subsidize the cost to make diapers more affordable in low-income and rural communities.

### Enhancing public awareness

Disseminating information to the public about the prevalence, etiology, and therapies for incontinence will effectively mitigate the stigma surrounding adult incontinence. Likewise, promoting community support through the organization of “community dialogues” that acknowledge cultural factors affecting the perception and management of adult incontinence in diverse communities will ameliorate the physical, psychological, social, and sexual well-being of women with UI, thus facilitating their rehabilitation and societal reintegration.

### Research collaboration

The government health institutions could foster research collaboration by establishing partnerships with academic institutions and industry stakeholders. Additionally, government could allocate resources towards research and development endeavors focused on innovative treatments, technologies, and assistive devices for managing incontinence. Furthermore, she could engage in collaborative efforts with international organizations like the World Health Organization (WHO) to gain access to valuable resources, guidelines, and best practices in adult incontinence management.

## Conclusion

There is a necessity for further research on the impact of urinary incontinence in SSA and the promotion of awareness among women regarding the medical and surgical options for urinary incontinence. The scarcity of urogynaecologist is a critical issue in low and middle-income countries of sub-Saharan Africa. The government in these regions should put in more effort to train a skilled workforce and provide the necessary facilities for managing this condition at a subsidized cost.

## Author contributions

CO: Writing – original draft, Writing – review & editing. CA: Writing – review & editing, Conceptualization.
